# Tubastatin ameliorates pulmonary fibrosis by targeting the TGFβ-PI3K-Akt pathway

**DOI:** 10.1371/journal.pone.0186615

**Published:** 2017-10-18

**Authors:** Shigeki Saito, Yan Zhuang, Bin Shan, Svitlana Danchuk, Fayong Luo, Martina Korfei, Andreas Guenther, Joseph A. Lasky

**Affiliations:** 1 Department of Medicine, Section of Pulmonary Diseases, Critical Care and Environmental Medicine, Tulane University Health Science Center, New Orleans, LA, United States of America; 2 Louisiana Clinical and Translational Science Center (LACaTS) Roadmap Scholars Program, New Orleans, LA, United States of America; 3 Department of Biomedical Sciences, Washington State University-Spokane College of Medical Sciences, Spokane, WA, United States of America; 4 Division of Cardiovascular Medicine, Department of Medicine, University of Missouri School of Medicine, Columbia, MO, United States of America; 5 Department of Biochemistry and Molecular Biology, University of Texas Medical School at Houston, Houston, TX, United States of America; 6 Department of Internal Medicine, Universities of Giessen and Marburg Lung Center (UGMLC), Justus-Liebig-University Giessen, Giessen, Germany; 7 Agaplesion Lung Clinic Waldhof Elgershausen, Greifenstein, Germany; University of Alabama at Birmingham, UNITED STATES

## Abstract

**Background:**

Idiopathic pulmonary fibrosis (IPF) is a chronic, progressive and fatal disease. Histone deacetylase 6 (HDAC6) alters function and fate of various proteins via deacetylation of lysine residues, and is implicated in TGF-β1-induced EMT (epithelial-mesenchymal transition). However, the role of HDAC6 in pulmonary fibrosis is unknown.

**Methods:**

HDAC6 expression in IPF and control lungs was assessed by quantitative real-time PCR (qRT-PCR) and immunoblots. Lung fibroblasts were treated with TGF-β1 ± HDAC6 inhibitors (Tubacin, Tubastatin, ACY1215, or MC1568), and fibrotic markers such as type I collagen were assessed using qRT-PCR and immunoblots. Mice were treated with bleomycin (oropharyngeal aspiration; single dose) ± Tubastatin (intraperitoneally injection; daily for 21 days), and lung collagen expression was gauged using immunoblots and trichrome staining. In a separate experiment, HDAC6 wild-type (WT) and knockout (KO) mice were administered bleomycin, and lungs were evaluated in the same manner.

**Results:**

HDAC6 expression was deregulated in IPF lungs. Among the HDAC6 inhibitors tested, only Tubastatin significantly repressed TGF-β1-induced expression of type-1 collagen in lung fibroblasts, and this finding was coupled with decreased Akt phosphorylation and increased Akt-PHLPP (PH domain and Leucine rich repeat Protein Phosphatase) association. Tubastatin repressed TGF-β1-induced S6K phosphorylation, HIF-1α expression, and VEGF expression. Tubastatin also repressed TGF-β1-induced inhibition of LC3B-II (a marker of autophagosome formation). In bleomycin-treated mouse lungs, HDAC6 expression was increased, and Tubastatin repressed type-1 collagen expression. However, in HDAC6 KO mice, bleomycin-induced type-1 collagen expression was not repressed compared to WT mice. Knockdown of HDAC6, as well as HDAC10, another potential Tubastatin target, did not inhibit TGF-β1-induced collagen expression in lung fibroblasts.

**Conclusions:**

HDAC6 expression is altered during lung fibrogenesis. Tubastatin represses TGF-β1-induced collagen expression, by diminishing Akt phosphorylation and regulating downstream targets such as HIF-1α-VEGF axis and autophagy. Tubastatin-treated WT mice are protected against bleomycin-induced fibrosis, but HDAC6 KO mice are not. Our data suggest that Tubastatin ameliorates pulmonary fibrosis, by targeting the TGFβ-PI3K-Akt pathway, likely via an HDAC6-independent mechanism.

## Background

Idiopathic pulmonary fibrosis (IPF) is a chronic, progressive and fatal disease of unclear etiology [1]. A prominent pathological feature of IPF is the formation of fibroblast foci, which consist of myofibroblasts and the extracellular matrix which they produce. Myofibroblasts are the principle effector cells synthesizing pro-fibrotic proteins such as α-smooth muscle actin (α-SMA), type-1 collagen, and fibronectin. Although multiple types of cells can differentiate into myofibroblasts, fibroblast to myofibroblast differentiation (FMD) is considered to be the major source for myofibroblast accumulation [2]. Some manuscripts suggest that epithelial-mesenchymal transition (EMT) is another source of myofibroblast accrual, although the contribution of EMT to pulmonary fibrosis remains controversial [3]. Among many fibrogenic cytokines implicated in the pathogenesis of pulmonary fibrosis, transforming growth factor (TGF)-β1 has been shown to play a crucial role. TGF-β1 induces FMD by activating Smad3 and Akt signaling pathways [4–6].

Histone deacetylases (HDACs) catalyze the removal of acetyl groups from lysine residues of both histone and nonhistone proteins. Deacetylation of histone tails regulates chromatin structure and transcription, whereas deacetylation of nonhistone proteins controls diverse cellular processes, such as cell signaling, cell motility, cell survival, protein degradation, and inflammation. HDAC inhibitors are being evaluated as therapeutic agents against cancer and many other diseases including fibrotic diseases [7–9]. We and others have shown that HDAC6, a class II HDAC, mediates TGF-β1-induced epithelial-mesenchymal transition (EMT) in A549 cells [10, 11]. We have also shown that IPF lungs exhibit distinct expression patterns of HDACs, including HDAC6, whose expression was elevated in type-II alveolar epithelial cells (AECII) and in myofibroblasts within fibroblast foci [12].

Although a few studies investigated the effect of non-selective pan-HDAC inhibitors as well as class I HDAC inhibitors on pulmonary fibrosis [12–15], the effects of selective inhibition of HDAC6 on pulmonary fibrosis has never been reported.

Therefore, we set out to investigate whether inhibition of HDAC6 can attenuate pulmonary fibrosis in experimental models. We observed that Tubastatin, a “selective” HDAC6 inhibitor, repressed TGF-β1-induced expression of type-1 collagen in lung fibroblasts, by repressing Akt phosphorylation and regulating downstream targets such as HIF-1α-VEGF axis and autophagy. Tubastatin also repressed bleomycin-induced type-1 collagen expression in mouse lungs. However, bleomycin-induced type-1 collagen expression in the lungs of HDAC6 KO mice did not appear different than lungs of WT littermates. These data suggest that Tubastatin ameliorates pulmonary fibrosis, by targeting the TGFβ-PI3K-Akt pathway, likely via an HDAC6-independent mechanism.

## Methods

### Reagents and antibodies

Tubacin, Tubastatin, and MC1568 were purchased from Sigma-Aldrich (St.Louis, MO). ACY1215 was purchased from ChemieTek　(Indianapolis, IN). Human recombinant TGF-β1 was purchased from R&D systems (Minneapolis, MN). Bleomycin was purchased from TEVA Pharmaceutical Industries (Petach Tikva, Israel). The following primary antibodies were purchased from the following companies: Santa Cruz (Dallas, TX; HDAC6 [H-300], HIF-1α), Abcam (Cambridge, MA; type-1 collagen), Sigma-Aldrich (α-SMA, acetylated α-tubulin), Cell Signaling Technology (Danvers, MA; β-actin, Smad2, phosphorylated Smad2, Smad3, phosphorylated Smad3, Akt, phosphorylated Akt (Ser473), PHLPP, Erk, phosphorylated Erk, p38, phosphorylated p38, HDAC6 [for the mouse samples], α-tubulin, LC3B). Secondary antibodies used were anti-mouse or anti-rabbit IgG HRP-linked antibodies (Cell Signaling Technology).

### Human lung tissue samples

Frozen lung tissue samples of patients with IPF (n = 20) and their controls (n = 10) were obtained from the Lung Tissue Research Consortium (LTRC), a program sponsored by the National Heart, Lung and Blood Institute. The clinical data and specimens had been de-identified by the LTRC. Additional lung tissue samples from patients with sporadic IPF (n = 9) and non-diseased control subjects (n = 8), collected in frame of the European IPF registry (eurIPFreg) were provided by the UGMLC Giessen Biobank (member of the DZL Platform Biobanking). Following lung homogenization, proteins and RNA were extracted in RIPA buffer (Cell Signaling Technology) and Trizol (Life Technologies; Carlsbad, CA), respectively, according to the manufacturers' protocols [16].

### Isolation of primary human lung fibroblasts

Primary human lung fibroblasts were isolated from explanted IPF and control lungs as described previously [12]. Experiments were carried out with IPF/control fibroblasts between passages 3 and 4.

### Cell culture of human lung fibroblasts

Normal human lung fibroblasts (NHLFs) were purchased from Lonza (Allendale, NJ) and maintained in fibroblast growth medium 2 (FGM-2; Lonza) for experiments until passage 6 per the provider’s instruction. Prior to treatment, cells that had reached 80% confluence were serum starved in fibroblast basal medium 2 (FBM-2; Lonza) with 0.2% bovine serum albumin (BSA) overnight [17].

### MTT assay

An MTT (3-(4,5-dimethylthiazol-2-yl)-2,5-diphenyltetrazolium bromide) assay was performed using an in vitro toxicology assay kit (Sigma-Aldrich) as described previously [17].

### RNA interference

RNA interference was carried out with HDAC6 siRNA (Gene Solution FlexiTube) and a negative control siRNA (All Star Negative), which were purchased from Qiagen (Hilden, Germany). For these experiments, 100 pmol siRNA oligo was transfected into NHLFs using Lipofectamine RNAiMAX reagent (Invitrogen; Waltham, MA), according to the manufacturer's protocol.

### Immunoblots

Cells were harvested using 1x RIPA buffer (Cell Signaling Technology) with protease inhibitor (cOmplete Mini, EDTA-free, Roche (Basel, Switzerland)) and phosphatase inhibitors (Phosphatase Inhibitor Cocktail 2 &3, Sigma-Aldrich; PMSF, Roche). Twenty μg of protein per sample was loaded onto NuPAGE Novex Bis-Tris 4–12% Protein Gels (Invitrogen) for electrophoresis and then transferred onto polyvinylidene difluoride (PVDF) membranes (0.45 μm, Millipore (Darmstadt, Germany)). Membranes were blocked in 5% non-fat dry milk (BioRad Laboratories (Hercules, CA)) for 1 hour at room temperature and then incubated with appropriate primary antibodies overnight at 4°C. Secondary antibodies and an ECL kit from GE Healthcare Life Sciences (Pittsburgh, PA) were employed to generate chemiluminescent signals. All immunoblot data represent triplicate repeats. Densitometry analysis was performed using National Institutes of Health (NIH) ImageJ software [17].

### Real time quantitative PCR

**Fig 1:**
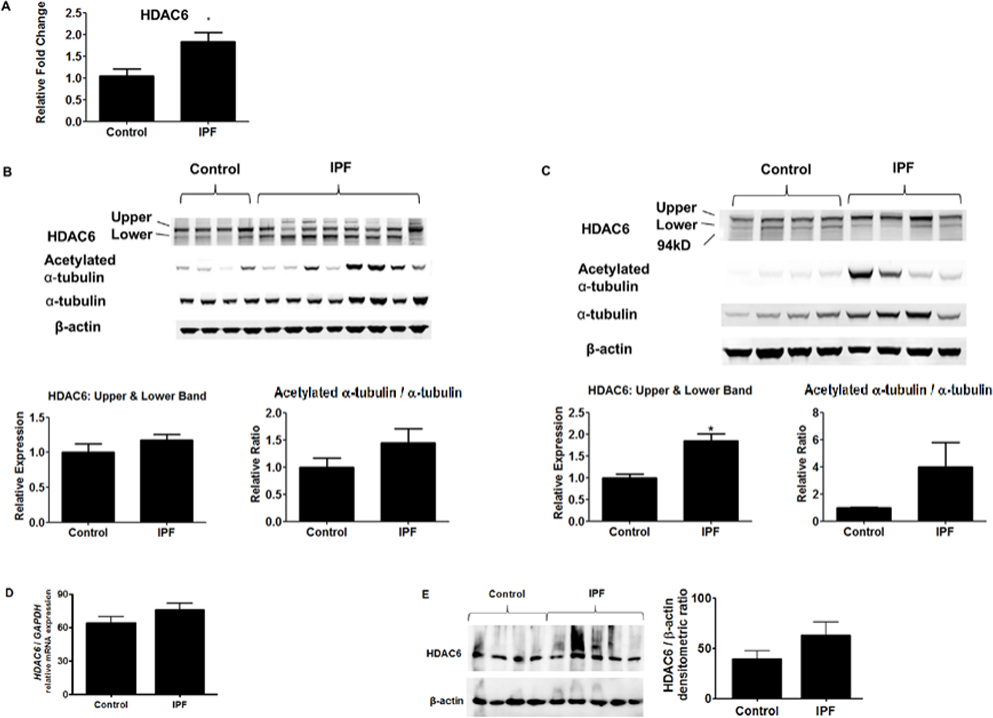
HDAC6 expression in IPF lungs. (A) qRT-PCR of lung homogenates of the US samples. * p<0.05 compared to the control. HDAC6 mRNA expression is increased in IPF lungs. (B, C) Immunoblots of lung homogenates of the US samples (B) and the European samples (C). The results of densitometry analyses are shown in the bar graphs. * p<0.05 compared to the control. HDAC6 protein expression appears altered in IPF; its expression is significantly increased in the European samples of IPF lungs, when the upper and lower bands are combined and analyzed together. Levels of acetylation of α-tubulin (a surrogate marker of HDAC6 activity) were not significantly altered in IPF, albeit there was a trend for increased acetylation in IPF lungs of both the US and European patient cohorts. (D) qRT-PCR of primary fibroblasts isolated from IPF lungs and their controls. There was a trend for increased HDAC6 mRNA expression in IPF fibroblasts, although the difference was not statistically significant (p = 0.155). (E) Immunoblots of primary fibroblasts isolated from IPF lungs and their controls. The results of densitometry analyses are shown in the bar graphs. There was a trend for increased HDAC6 protein expression in IPF fibroblasts, although there was not a statistically significant difference (p = 0.109).

Real time quantitative PCR was performed using the iCycler (Bio-Rad), and SYBR green supermix (Bio-Rad) was employed according to the manufacturer’s instructions, along with gene-specific primers. The specific gene's cycle threshold (Ct) values were normalized to 36B4 and compared with the control group that was assigned a value of 1 to calculate the relative fold change in expression as previously described [18]. The results represented three independent experiments. Primer sequences were: h-Collagen-1: Fwd: CGGAGGAGAGTCAGGAAGG, Rev: CACAAGGAACAGAACAGAACA; m-Collagen-1: Fwd: GCCAAGAAGACATCCCTGAAG, Rev: CACAAGGAACAGAACAGAACAG; h-α-SMA: Fwd: GAAGAAGAGGACAGCACT, Rev: TCCCATTCCCACCATCAC; 36B4: Fwd: CGACCTGGAAGTCCAACTAC; Rev: ATCTGCTGCATCTGCTTG; h-HDAC6: Fwd: CAACTGAGACCGTGGAGAG, Rev: CCTGTGCGAGACTGTAGC. Gene specific primers for h-HDAC6 and h-GAPDH used for Fig 1D are described in [12].

### Mouse experiments with bleomycin and Tubastatin

C57/BL6 male mice were purchased from Charles River. HDAC6 knockout (KO) mice (C57/BL6 background) are kind gifts of Dr. Tso-Pang Yao (Duke University). For experiments using Tubastatin, 6–10 week old male mice (Charles River Laboratories; Wilmington, MA) were given bleomycin (2units/kg; dissolved in 50ul PBS) or the same amount of PBS by oropharyngeal aspiration on Day 0, which was followed by daily intraperitoneal injection of Tubastatin (80 mg/kg/day) or the same amount of vehicle (DMSO) on Day 1 through Day 21 (n = 4-5/group). Mice were sacrificed on Day 21. Left lungs were perfused with 10% formalin and paraffin-embedded, and then tissue slides (5μm thickness) were prepared for Masson’s trichrome staining. Right lungs were harvested and homogenized in liquid nitrogen for immunoblot analysis. For experiments using HDAC6 KO mice, 6–10 week old male KO mice and their wild-type (WT) male littermates were used. Mice were sacrificed on Day 21 and lungs were analyzed in the manner described above. The mice were weighed daily, and were euthanized by carbon dioxide asphyxiation if there were any signs of severe suffering or distress (e.g. inability to rise or ambulate, weight loss >20%, labored breathing, distended abdomen, hunched posture and ruffled fur).

### Isolation of mouse lung fibroblasts (MLFs)

Primary cultures of MLFs isolated from HDAC6 WT or KO mice were established as previously described [19]. MLFs were maintained in DMEM (Gibco; Waltham, MA) supplemented with 10% fetal bovine serum (FBS) for experiments until passage 3. Prior to the treatment, they were serum starved in DMEM with 0.2% BSA overnight.

### Statistical analysis

Data are expressed as the mean±standard error. Unless otherwise indicated, statistical analyses for multiple group comparison were conducted using one-way analysis of variance followed by the Bonferroni post hoc test (GraphPad Prism, Version 5; La Jolla, CA). P value < 0.05 was considered significant.

## Results

### HDAC6 expression is altered in IPF lungs

We first examined the level of HDAC6 expression in lung tissue homogenates from individuals with IPF and individuals without IPF. Patient demographics are shown in S1 Table. HDAC6 mRNA expression was mildly increased in IPF lungs of the US samples (Fig 1A), which was consistent with our previous finding from the European samples [12]. HDAC6 protein expression was also altered in IPF lung homogenates of both the US and European patient cohorts, although the western blots showed different patterns of immunoreactive bands between these samples (Fig 1B and 1C). When the two major bands near the estimated size of HDAC6 protein (131 kDa) were analyzed together, there was a trend for increased HDAC6 protein expression in the US IPF samples (Fig 1B), and there was a significant increase in HDAC6 protein expression in the European IPF samples (Fig 1C). Levels of acetylation of α-tubulin (a surrogate marker of HDAC6 activity) were not altered significantly in IPF compared to control lungs, albeit there was a trend for increased acetylation in IPF lungs of both the US and European patient cohorts.

We also examined HDAC6 expression in fibroblasts isolated from IPF and control lungs. There was a trend for increased HDAC6 mRNA and protein expression in fibroblasts isolated from IPF lungs compared to their controls, although there was no statistically significant difference (Fig 1D and 1E). We have previously shown that the acetylation status of α-tubulin was significantly reduced in IPF fibroblasts, compared to their controls [12].

### α-tubulin acetylation is reduced in TGF-β1-treated NHLFs

**Fig 2:**
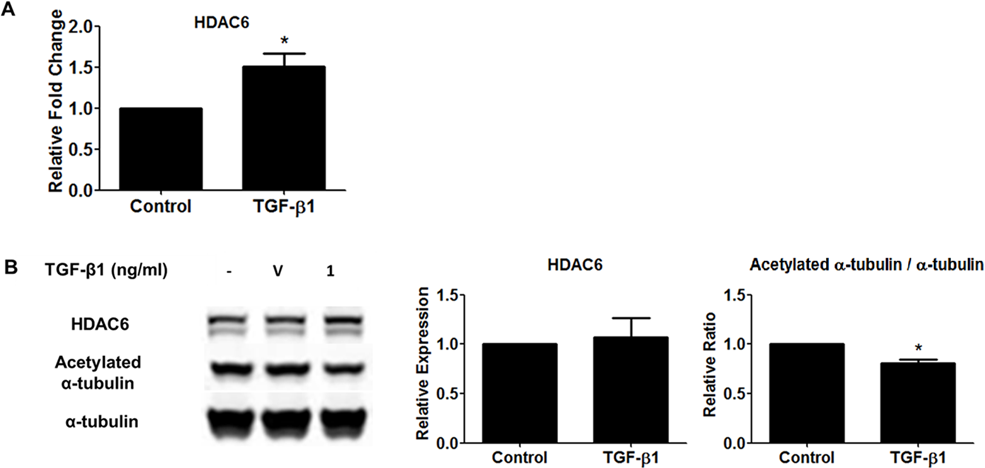
HDAC6 expression in normal human lung fibroblasts (NHLFs) treated with TGF-β1. Subconfluent NHLFs were serum-starved overnight and then treated with TGF-β1 (1 ng/ml). (A) The level of HDAC6 gene expression was assessed using qRT-PCR at 24 hours. * p<0.05 compared to the control. HDAC6 mRNA expression is increased in TGF-β1-treated NHLFs. (B) The levels of HDAC6 protein expression and α-tubulin acetylation were assessed using immunoblots at 48 hours. V denotes vehicle. The results of densitometry analyses are shown in the bar graphs. * p<0.05 compared to the control. TGF-β1 does not significantly increase HDAC6 protein expression, but significantly reduces α-tubulin acetylation level, suggesting that HDAC6 activity may be increased by TGF-β1.

Next we investigated the effect of TGF-β1 on HDAC6 expression and deacetylase activity in NHLFs. TGF-β1 only mildly increased HDAC6 mRNA expression, and did not significantly increase HDAC6 protein expression. However, a small but statistically significantly reduction in α-tubulin acetylation was detected in response to TGF-β1 (Fig 2). This was consistent with our previous finding that IPF fibroblasts showed reduced α-tubulin acetylation [12], and suggests that TGF-β1 may increase HDAC6 activity (or modulate activities of other α-tubulin acetylating/deacetylating enzymes such as α-tubulin acetyltransferase-1 (αTAT-1) and sirtuin 2 (SIRT2))[20].

### Tubastatin inhibits TGF-β1-induced collagen expression in lung fibroblasts

**Fig 3:**
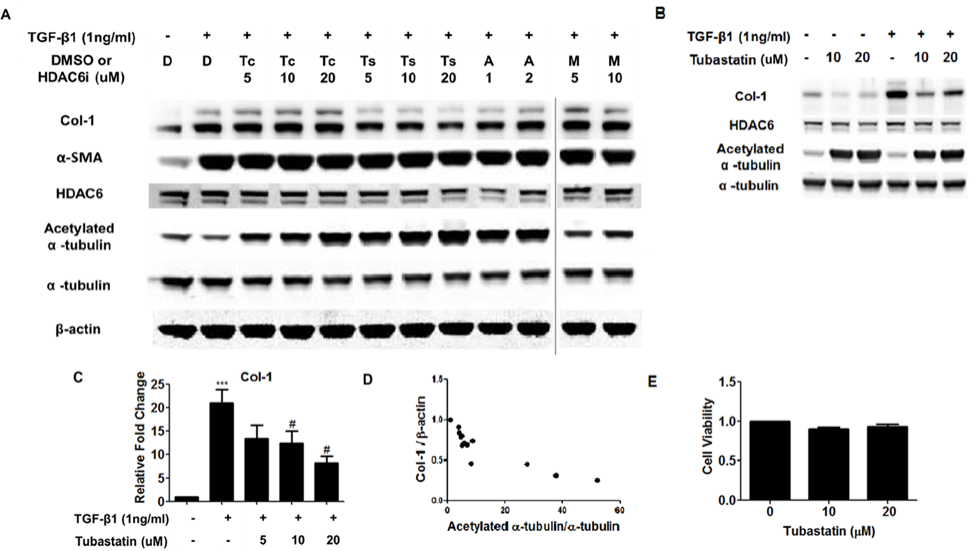
Tubastatin decreases TGF-β1-induced expression of type-1 collagen. (A-E) Subconfluent NHLFs (A, C, D, E) or IPF fibroblasts (B) were pretreated with an HDAC6 inhibitor (Tubacin, Tubastatin, ACY1215, or MC1568) for 6 hours and then co-treated with TGF-β1 and the HDAC6 inhibitor. (A, B) Protein expression levels were assessed using immunoblots at 48 hours. D: DMSO, Tc: Tubacin, Ts: Tubastatin, A: ACY1215, M: MC1568; Col-1: type-1 collagen, α-SMA: α-smooth muscle actin (C) Gene expression levels for collagen-1 (col1a1) were assessed using qRT-PCR at 24 hours. * p<0.05 compared to the control; # p<0.05 compared to the TGF-β1. (D) Correlation between the degree of α-tubulin acetylation and collagen-1 protein expression in experiments involving Tubastatin treatment. There was a modest inverse correlation between the degree of α-tubulin acetylation and collagen-1 expression (r^2^ = 0.7602 [p<0.0001] for pooled results of six independent experiments.). (E) An MTT assay showing that Tubastatin does not affect NHLF viability. Values for cell viability were normalized to the control group (= DMSO treatment).

We investigated whether HDAC6 inhibition could affect TGF-β1-induced FMD. We tested two traditional “selective” HDAC6 inhibitors, Tubacin (the 1^st^ generation) and Tubastatin (the 2^nd^ generation; traditionally thought to be more selective and potent than Tubacin, although some recent studies questioned this notion [21, 22]), as well as ACY1215 (a newer HDAC6 inhibitor), and MC1568 (a class II HDAC inhibitor). Compared to Tubacin, Tubastatin and ACY1215 induced more robust hyperacetylation of α-tubulin. Tubastatin (and ACY1215, to less degree), but not Tubacin or MC1568, significantly repressed TGF-β1-induced expression of type-1 collagen in NHLFs and IPF fibroblasts (Fig 3A, 3B and 3C; S1 Fig). There was a modest inverse correlation between the degree of α-tubulin acetylation and type-1 collagen protein expression (r^2^ = 0.7602, p p<0.0001), suggesting that inhibition of HDAC6’s deacetylase activity and/or hyperacetylation of α-tubulin may reduce TGF-β1-induced type-1 collagen expression (Fig 3D). An MTT assay revealed no significant differences in cell viability when cells were exposed to the employed concentrations of Tubastatin (Fig 3E).

### Tubastatin decreases TGF-β1-induced phosphorylation of Akt, likely by restoring the association of Akt and PHLPP

**Fig 4:**
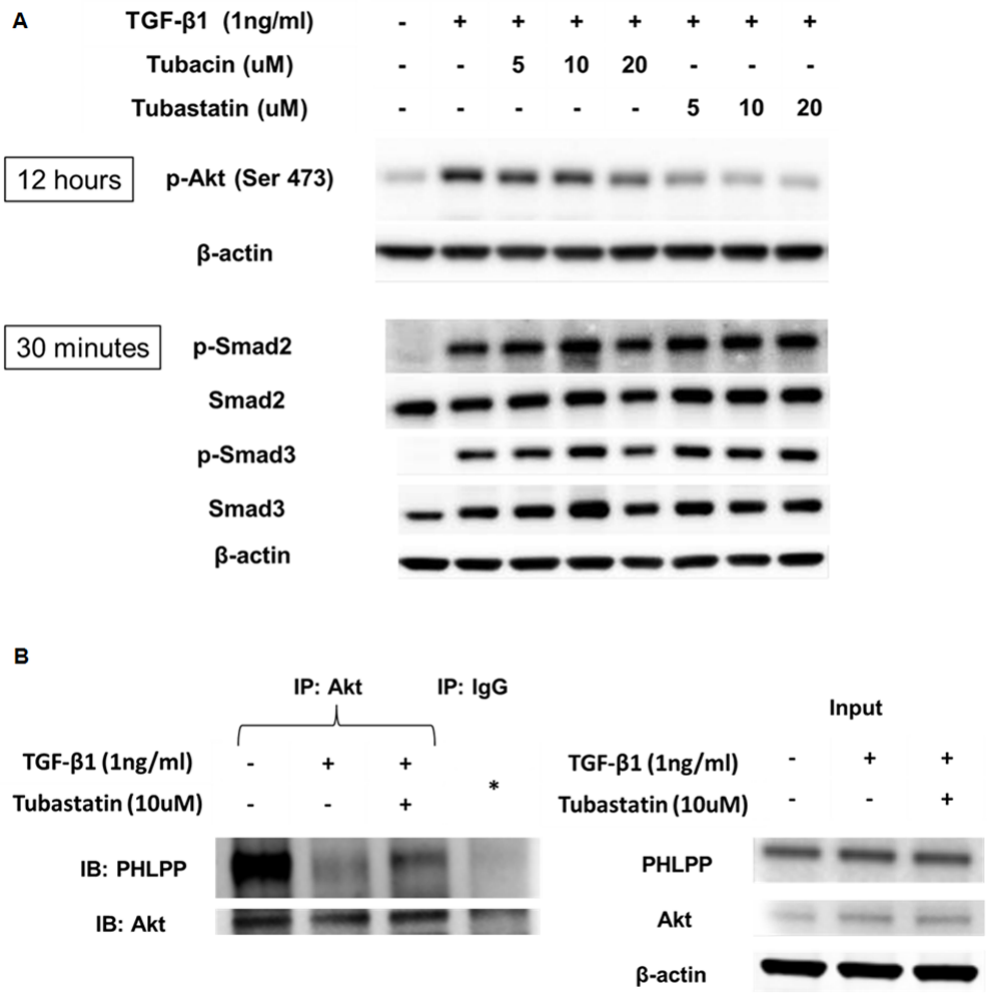
Tubastatin decreases TGF-β1-induced phosphorylation of Akt, likely by restoring the association of Akt and PHLPP. (A) Subconfluent NHLFs were pretreated with Tubastatin for 6 hours and then co-treated with TGF-β1 and Tubastatin. Protein expression levels were assessed using immunoblots at the designated time points. Tubastain repressed TGF-β1-induced phosphorylation of Akt, but not Smad2 or Smad3. (B) Subconfluent NHLFs were pretreated with Tubastatin for 6 hours and then co-treated with TGF-β1 and Tubastatin for 12 hours. *Left*: Cell lysates were immunoprecipitated with an anti-Akt antibody, and protein expression levels for PHLPP and Akt were assessed using immunoblots. IP denotes immunoprecipitation. The fourth lane (*) represents IP using isotype control IgG antibody and the combined samples from the three treatment groups (i.e. an equal amount of the sample from each treatment group was mixed). *Right*: “Input” refers immunoblots without IP. TGF-β1 disrupted Akt-PHLPP association, and this was ameliorated by Tubastatin.

Next we set out to identify the molecular mechanism by which Tubastatin represses TGF-β1-induced type-1 collagen expression. First, we checked phosphorylation of Smad 2 and 3 (canonical pathway) as well as p38 MAPK and ERK (non-canonical pathway), and found no difference in their phosphorylation with or without Tubastatin (Fig 4; S2 Fig). Next, we assessed phosphorylation of Akt, because repressing Akt phosphorylation by inhibiting PI3K activity and/or enhancing PTEN activity / expression has been associated with reduced collagen I expression in lung fibroblasts [23–25]. We found that Tubastatin inhibits phosphorylation of Akt at serine (Ser) 473 (Fig 4A). We also found that TGF-β1 decreased the association between Akt and PHLPP (phosphatase responsible for Ser 473 dephosphorylation of Akt) and observed that Tubastatin partially restored the Akt-PHLPP association (Fig 4B). The expression of PHLPP and other phosphatases (such as PTEN, PP1, and PP2A) did not increase with Tubastatin treatment (S3 Fig). These data suggest that Tubastatin represses TGF-β1-induced type-1 collagen expression, likely by restoring Akt-PHLPP association and promoting Akt dephosphorylation.

### Tubastatin represses TGF-β1-induced phosphorylation of S6K (downstream of Akt-mTORC1) and subsequent HIF-1α expression

**Fig 5:**
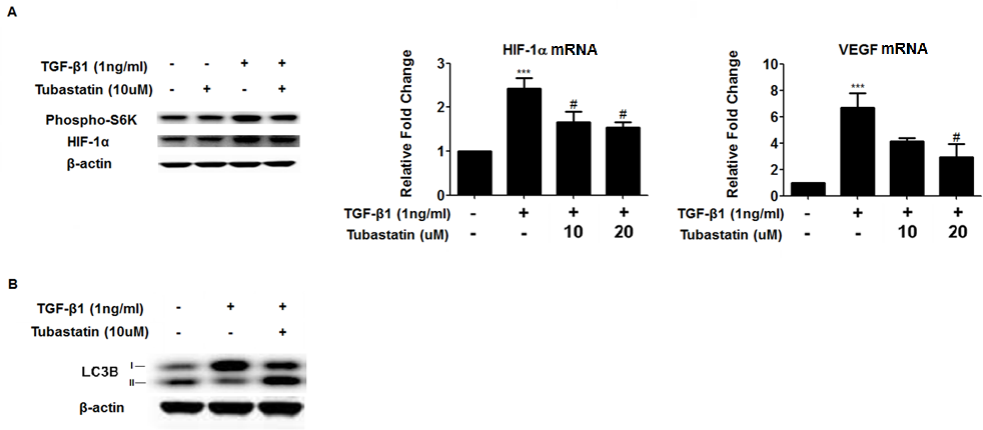
Tubastatin counteracts with the effects of TGF-β1 on HIF-1α-VEGF axis and autophagy. (A-B) Subconfluent NHLFs were pretreated with Tubastatin for 6 hours, and then co-treated with TGF-β1 and Tubastatin for 24 hours (A) or 48 hours (B). (A) *Left*; Protein expression levels were assessed using immunoblots. Phospho- S6K denotes phosphorylated p70 ribosomal protein S6 kinase. *Middle and Right*; Gene expression levels for HIF-1α and VEGF were assessed using qRT-PCR. *** p<0.0005 compared to the control; # p<0.05 compared to the TGF-β1. Tubastatin decreases TGF-β1-induced phosphorylation of S6K and expression of HIF-1α and VEGF. (B) Protein expression levels were assessed using immunoblots. Tubastatin represses TGF-β1-induced inhibition of LC3B-II.

Next we examined downstream of Akt pathway, to further explore the mechanism by which Tubastatin represses TGF-β1-induced gene and protein expression of type-1 collagen. First we measured levels of phosphorylated p70 ribosomal protein S6 kinase (S6K) and HIF-1α, because 1) mTORC1, which is downstream of Akt, phosphorylates S6K and eukaryotic translation- initiation factor 4E (eIF4E)-binding protein 1 (4EBP1) to activate translation of proteins, such as hypoxia-inducible factor (HIF)-1α [26, 27], and 2) HIF-1α has been shown to regulate collagen expression [28]. We found that Tubastatin repressed TGF-β1-induced phosphorylation of S6K and subsequent HIF-1α protein expression. Furthermore, Tubastatin repressed TGF-β1-induced mRNA expression of vascular endothelial growth factor (VEGF; a well-known HIF-1α target gene) as well as HIF-1α (Fig 5A).

### Tubastatin represses TGF-β1-induced reduction of LC3B-II (a marker of autophagosome formation)

Next, we checked the level of LC3B-II, a marker of autophagosome formation, because reduced Akt activity and subsequent upreguation of autophagy have been shown to lead to decreased collagen accumulation [29]. We found that TGF-β1 decreased LC3B-II (which is suggestive of reduced autophagosome formation and is consistent with the previous reports [30, 31]) and that Tubastatin repressed TGF-β1-induced reduction of LC3B-II (Fig 5B). Taken together, out data suggest that repression of TGF-β1-induced Akt phosphorylation by Tubastatin leads to repression of HIF-1α-VEGF axis and induction of autophagy, which results in repression of TGF-β1-induced collagen expression.

### Tubastatin-treated WT mice, but not HDAC6 KO mice, are protected against bleomycin-induced pulmonary fibrosis

**Fig 6:**
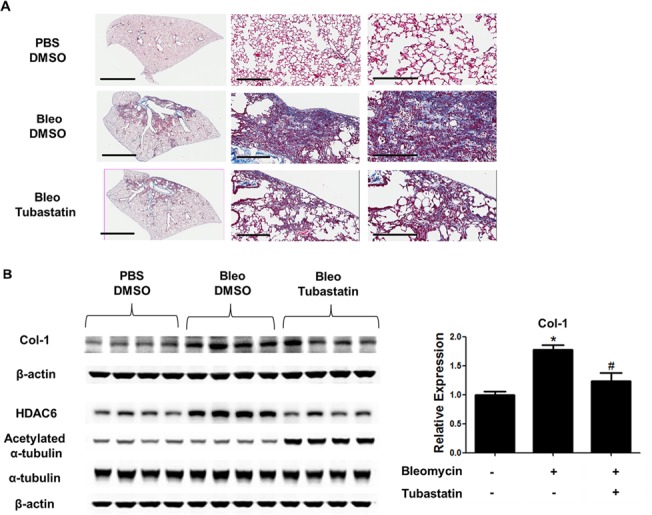
Tubastatin-treated mice are protected against bleomycin-induced pulmonary fibrosis. Mice were treated with PBS or bleomycin (2units/kg; by oropharyngeal aspiration) on Day 0, followed by daily intraperitoneal injection of DMSO or Tubastatin (80 mg/kg/day) on Day 1 through Day 21 (n = 4-5/group). (A) Collagen expression was assessed using trichrome staining of lung sections. Representative images (taken at 40x magnification) are shown. Scale bars: 3mm (left panels), 300μm (middle panels), 200μm (right panels). (B) Protein expression levels were assessed using immunoblots of lung homogenates. Densitometry analysis of type-1 collagen expression is shown in the bar graphs. * p<0.05 compared to the control (= PBS+DMSO); # p<0.05 compared to Bleomycin+DMSO.

**Fig 7:**
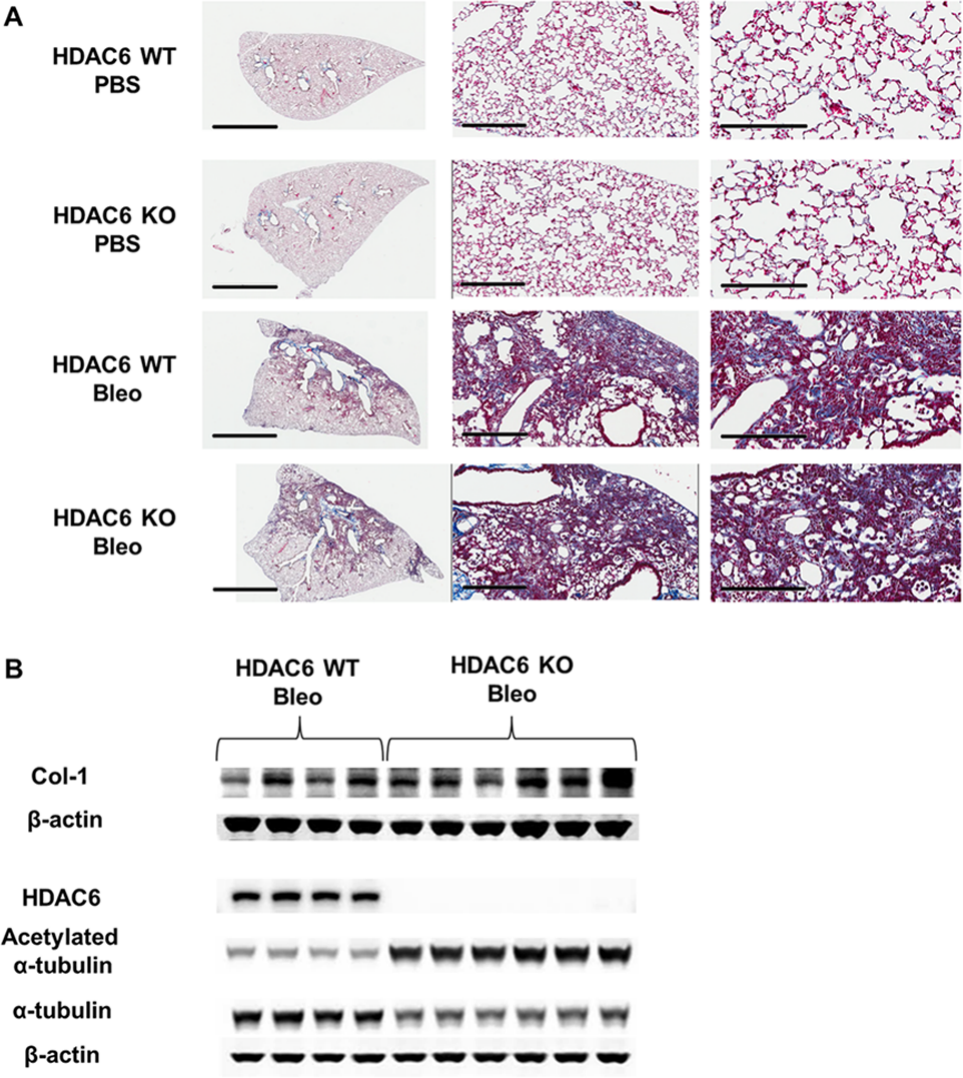
HDAC6 knockout mice are not protected against bleomycin-induced pulmonary fibrosis. (A, B) HDAC6 wild-type (WT) and knockout (KO) mice were treated with PBS or bleomycin (2units/kg; by oropharyngeal aspiration) on Day 0 and then lungs were harvested on Day 21. (A) Collagen expression was assessed using trichrome staining of lung sections. Representative images (taken at 40x magnification) are shown. Scale bars: 3mm (left panels), 300μm (middle panels), 200μm (right panels). (B) Protein expression levels were assessed using immunoblots of lung homogenates.

We investigated the role of HDAC6 and the effect of Tubastatin in a murine model of bleomycin-induced pulmonary fibrosis (Fig 6A and 6B). Bleomycin treatment increased HDAC6 protein expression, although it did not significantly alter the acetylation status of α-tubulin (Fig 6B). Tubastatin treatment significantly decreased HDAC6 protein expression, increased the level of acetylated α-tubulin, and ameliorated bleomycin-induced type-1 collagen expression (Fig 6A and 6B). However, bleomycin induced lung type-1 collagen to a similar extent in both WT and HDAC6 KO littermates (Fig 7A and 7B).

### Knockdown of HDAC6, as well as HDAC10, another potential Tubastatin target, does not inhibit TGF-β1-induced collagen in lung fibroblasts

**Fig 8:**
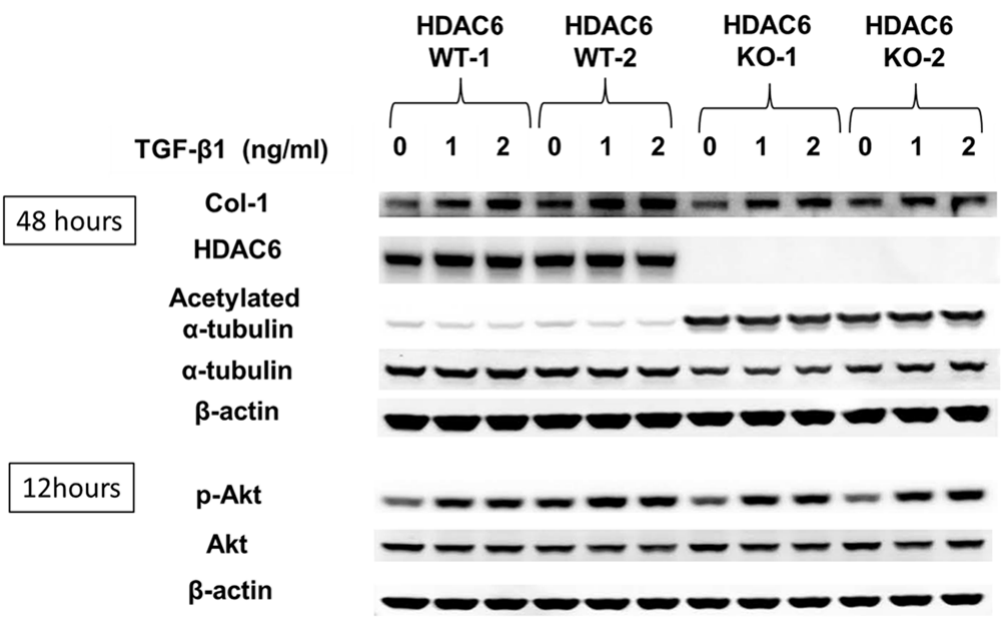
TGF-β1 induces type-1 collagen expression and Akt phosphorylation in HDAC6 WT and knockout mouse lung fibroblasts (MLFs), to a similar extent. Lung fibroblasts were isolated from two WT and two HDAC6 knockout mice. Cells were serum starved and then exposed to TGF-β1 (1ng/ml) for 48hrs (A) or 12 hours (B).

**Fig 9:**
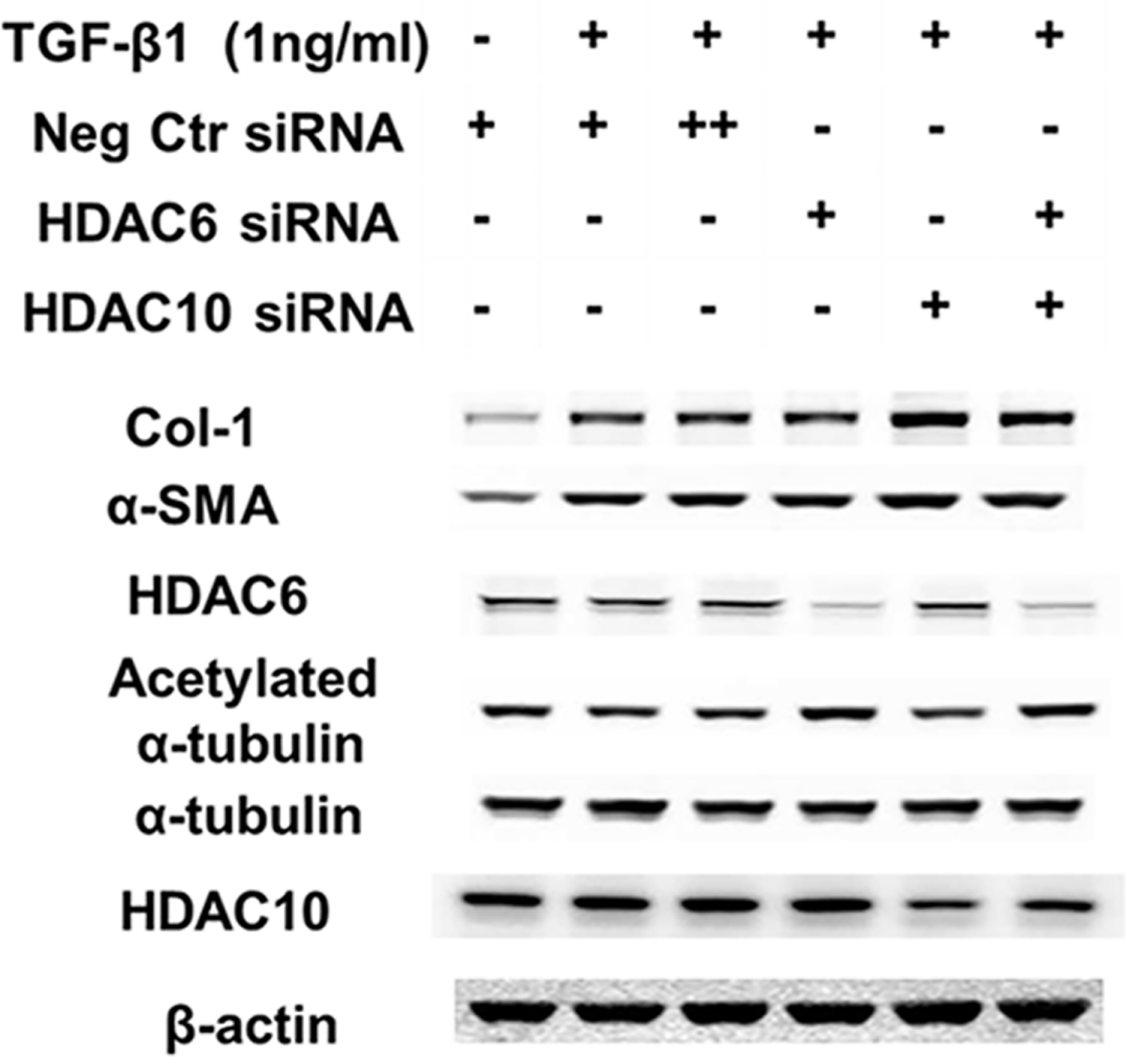
HDAC6 knockdown does not ameliorate TGF-β1-induced type-1 collagen expression in NHLFs. Subconfluent NHLFs were pretreated with siRNA for 24 hours and then co-treated with TGF-β1 for 48hours. ++ denotes double concentration. The immunoblots show that knockdown of HDAC6 and/or HDAC10 failed to repress TGF-β1-induced type-1 collagen expression in NHLFs.

Mouse lung fibroblasts (MLFs) isolated from HDAC6 WT and KO mice exhibited TGF-β1-induced type-1 collagen expression and Akt phosphorylation to a similar extent (Fig 8). HDAC6 knockdown using siRNA also failed to inhibit TGF-β1-induced expression of type-1 collagen in NHLFs. We also knocked down HDAC10, another potential Tubastatin target [21]. Neither HDAC10 knockdown or HDAC6/10 double-knockdown inhibited TGF-β1-induced expression of type-1 collagen in NHLFs (Fig 9).

## Discussion

This study addressed the potential role of HDAC6 in modulating lung fibrogenesis by examining HDAC6 expression in IPF lung homogenates and by examining the effect of HDAC6 inhibition on fibrogenesis in experimental models. We observed aberrant HDAC6 expression in IPF lungs. First, we confirmed our previous finding that HDAC6 mRNA expression is increased in IPF lungs [12]. Next, we found that there was a trend for increased HDAC6 protein expression in the US IPF samples and a significant increase in HDAC6 protein expression in the European IPF samples, when the two major bands near the estimated size of HDAC6 protein (131 kDa) were analyzed together. It is not clear why the band pattern of HDAC6 in the immunoblots appears different between the US-IPF samples and the European-IPF samples, but this may be due to limited number of samples. The lower bands may reflect the HDAC6 splicing variant p114 which we have previously reported [32], but may rather reflect post-translationally modified HDAC6, since the distance between the upper bands and the lower bands appears larger than the distance usually observed between p131 (normal variant) and p114 (splicing variant).

Levels of acetylation of α-tubulin, a surrogate marker of HDAC6 activity, were not significantly altered in IPF lungs in comparison to their controls, although there was a slight trend for increased acetylation in IPF. This was opposite to our expectation, because α-tubulin is expected to be less acetylated when HDAC6 expression is increased, and because α-tubulin acetylation is decreased in TGF-β1-treated NHLFs and IPF lung fibroblasts as well as TGF-β1-treated A549 cells [10]. This “discrepancy” may be solely due to large heterogeneity among human lung samples, but it may also be due to that α-tubulin acetylation is also regulated by αTAT-1, SIRT2 [20], and possibly HDAC3 [33]. Nevertheless, in our previous study, acetylation status of α-tubulin was reduced in fibroblasts isolated from IPF lungs, compared to fibroblasts isolated from control lungs [12].

We have shown that Tubastatin, a “selective” HDAC6 inhibitor, represses TGF-β1-induced type-1 collagen expression in lung fibroblasts. As for the mechanism, our in vitro studies demonstrated that Tubastatin represses TGF-β1-induced Akt phosphorylation. This is an important observation, because it has been shown that 1) PI3K-Akt pathway is activated in pulmonary fibrosis and 2) inhibition of this pathway inhibits FMD and ameliorates pulmonary fibrosis [28, 34]. In other words, targeting this pathway has been thought to have therapeutic potential, since repressing Akt phosphorylation by inhibiting PI3K activity and/or enhancing PTEN activity / expression leads to reduced fibrogenesis [23–25, 28, 34–36]. The PI3K-Akt pathway has been shown to contribute to pulmonary fibrosis via multiple mechanisms. For example, the PI3K-Akt pathway contributes to pulmonary fibrosis by inducing expression of HIF-1α and VEGF. HIF-1α is elevated in pulmonary fibrosis [37] and may contribute to disease progression, at least partially by inducing VEGF expression and subsequent collagen expression [28]. Our finding that Tubastatin repressed TGF-β1-induced expression of collagen, at least partially by repressing the PI3K-Akt-HIF-1α-VEGF pathway is consistent with these previous reports. The PI3K-Akt pathway also contributes to pulmonary fibrosis via impairing autophagy. For example, lung tissues from IPF patients demonstrated increased activity of mTORC1 (an Akt target) and decreased autophagy. Autophagy is not enhanced in IPF, despite elevations in major regulators of autophagy, such as endoplasmic reticulum (ER) stress, oxidative stress, and HIF-1α [30]. TGF-β1 inhibited autophagy in fibroblasts in vitro at least in part via activation of mTORC1 [30]. Rapamycin (which inhibits mTORC1) and other autophagy-inducing agents protect against fibrosis, at least partially by promoting autophagic degradation of extracellular proteins [30, 31, 38]. Our finding that Tubastatin repressed TGF-β1-induced collagen expression, at least partially by repressing the PI3K-Akt pathway and subsequently by inducing autophagy (as suggested by increased LC3B-II, a marker of autophagosome formation), is consistent with these previous reports.

With regard to the mechanism of reduced Akt phosphorylation by Tubastatin, we found that Tubastatin decreases Akt phosphorylation at least in part by inhibiting TGF-β1-induced Akt-PHLPP dissociation. PHLPP is responsible for terminating Akt signaling by dephosphorylating Akt on serine 473, and its importance in various cellular processes has been increasingly recognized [39]. Further experiments to investigate the role of HDAC-Akt-PHLPP-axis in fibrogenesis are ongoing in our laboratory.

We have also shown that Tubastatin treatment reduces bleomycin-induced type-1 collagen expression in mice. In contrast, HDAC6 KO mice were not protected from bleomycin-mediated pulmonary fibrosis. Moreover, TGF-β1-induced type-1 collagen expression was not decreased in MLFs isolated from HDAC6 KO mice, as compared to MLFs isolated from HDAC6 WT mice. TGF-β1-induced type-1 collagen expression was not decreased in NHLFs treated with HDAC6 siRNA, as compared to NHLFs treated with negative control siRNA, either.

There are a few possible explanations as to the reason why Tubastatin treatment (inhibition of HDAC6 deacetylase activity) and HDAC6 knockdown/knockout lead to different results. The first possibility is that Tubastatin has off-target effects aside from HDAC6. This may be the most likely scenario, given that even high-dose Tubacin (which caused robust hyperacetylation of α-tubulin) failed to repress TGF-β1-induced type-1 collagen expression. For example, Tubastatin might inhibit other HDACs or other proteins. Recent studies suggested that Tubastatin may have some inhibitory effects against HDAC10 [21, 22]. However, knockdown of HDAC6 and/or 10 with siRNA did not inhibit TGF-β1-induced type-1 collagen expression in NHLFs (Fig 9). The second possibility is that the variant results are simply because pharmacologic inhibition of HDACs are not equivalent to genetic knockdown/knockout, especially in terms of effects on multiprotein complex formation and function [40]. HDAC6 has functions beyond its catalytic (deacetylating) activity. For example, HDAC6 harbors a C-terminal zinc finger domain (ZnF-UBP) through which HDAC6 binds to cytotoxic poly-ubiquitinated proteins and ferries them into autophagosomes for their degradation, thereby preventing protein aggregation [41]. In addition, HDAC6 negatively regulates NLRP3 inflammasome activation through its association with ubiquitinated NLRP3, likely by sequestrating the ubiquitinated NLRP3 from the formation of inflammasome assembly [42]. Therefore, it is plausible that loss of HDAC6’s ubiquitin-binding function aggravates pulmonary fibrosis by causing defective autophagy (as a result of deregulated aggresome formation) and/or activation of NLRP3 inflammasome [43, 44], whereas loss or inhibition of HDAC6’s deacetylating function ameliorates pulmonary fibrosis by hyperacetylating target proteins. In other words, it is tempting to speculate that inhibition of deacetylase function is protective against fibrosis whereas inhibition of both deacetylase and non-deacetylase function (e.g. ubiquitin-binding function) is not. Further studies to investigate this possibility are ongoing in our laboratory.

## Conclusions

In summary, this work demonstrates that HDAC6 expression is deregulated in IPF lungs and in a murine model of bleomycin-induced pulmonary fibrosis. Tubastatin, a “selective” HDAC6 inhibitor, inhibits TGF-β1-induced type-1 collagen expression in lung fibroblasts, and reduces type-1 collagen expression in murine bleomycin-induced pulmonary fibrosis. However, HDAC6 KO mice are not protected against bleomycin-induced lung injury. Our data suggests that Tubastatin ameliorates pulmonary fibrosis, by targeting the TGFβ-PI3K-Akt pathway, likely via an HDAC6-independent mechanism.

## Declarations

### Ethics approval and consent to participate

All protocols for animal studies were approved by the Institutional Animal Care and Use Committee of Tulane University. The UGMLC Giessen Biobank has been approved by the Ethics committee of the Justus-Liebig-University of Giessen (111/08 and 58/15).

### Consent for publication

Not applicable

### Funding

Supported by the Wetmore Foundation (Project: 553862, Task: M1, Award: 553860G1 [to JAL]; Project: 555007, Task: M1, Award: 555007G1 [to SS]) and by 1 U54 GM104940 from the National Institute of General Medical Sciences of the National Institutes of Health, which funds the Louisiana Clinical and Translational Science Center. The content is solely the responsibility of the authors and does not necessarily represent the official views of the National Institutes of Health.

## Supporting information

S1 Table Patient demographics: Donors of IPF lungs and control lungs. (XLSX)

S1 Fig Tubastatin decreases TGF-β1-induced expression of type-1 collagen. Subconfluent NHLFs were pretreated with an HDAC6 inhibitor (Tubacin, Tubastatin, ACY1215, or MC1568) for 6 hours and then co-treated with TGF-β1 and the HDAC6 inhibitor. Protein expression levels were assessed using immunoblots at 48 hours. Col-1 denotes type-1 collagen.
(TIF)

S2 Fig Tubastatin does not decrease TGF-β1-induced phosphorylation of MAPK. Subconfluent NHLFs were pretreated with Tubastatin for 6 hours and then co-treated with TGF-β1 and Tubastatin. Protein expression levels were assessed using immunoblots at 3 hours post treatment. Tubastatin did not decrease TGF-β1-induced phosphorylation of Erk or p38 MAPK.
(TIF)

S3 Fig Tubastatin-mediated Akt dephosphorylation is not due to alterations in the expression level of phosphatases involved in Akt dephosphorylation. Subconfluent NHLFs were pretreated with Tubastatin for 6 hours and then co-treated with TGF-β1 and Tubastatin for 12 hours.
(TIF)
