# Supplementary figures and images for: Tubastatin ameliorates pulmonary fibrosis by targeting the TGFβ-PI3K-Akt pathway
Source: PLoS One. 2017 Oct 18;12(10):e0186615. doi: 10.1371/journal.pone.0186615 (PMC5646855; doi:10.1371/journal.pone.0186615)

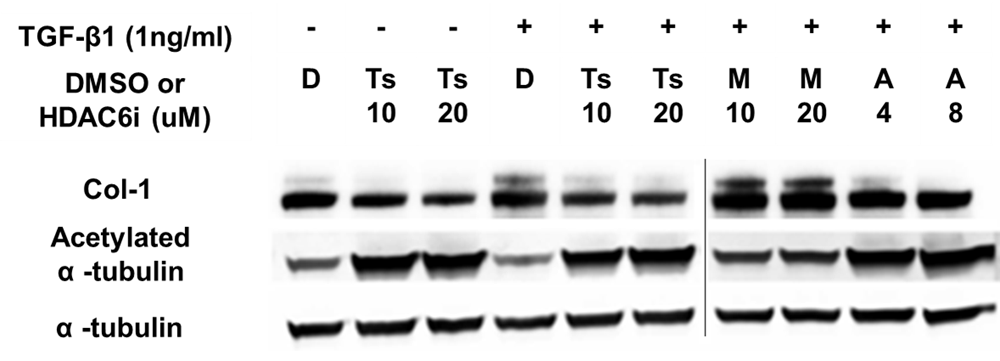

Supplement: S1 Fig — Subconfluent NHLFs were pretreated with an HDAC6 inhibitor (Tubacin, Tubastatin, ACY1215, or MC1568) for 6 hours and then co-treated with TGF-β1 and the HDAC6 inhibitor. Protein expression levels were assessed using immunoblots at 48 hours. Col-1 denotes type-1 collagen. (TIF) [file pone.0186615.s002.tif]

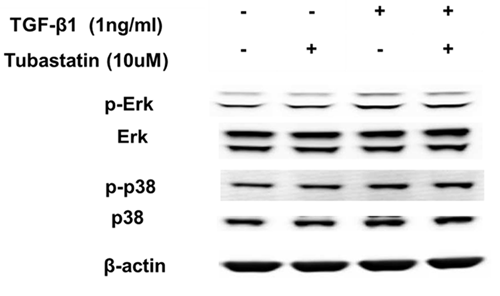

Supplement: S2 Fig — Subconfluent NHLFs were pretreated with Tubastatin for 6 hours and then co-treated with TGF-β1 and Tubastatin. Protein expression levels were assessed using immunoblots at 3 hours post treatment. Tubastatin did not decrease TGF-β1-induced phosphorylation of Erk or p38 MAPK. (TIF) [file pone.0186615.s003.tif]

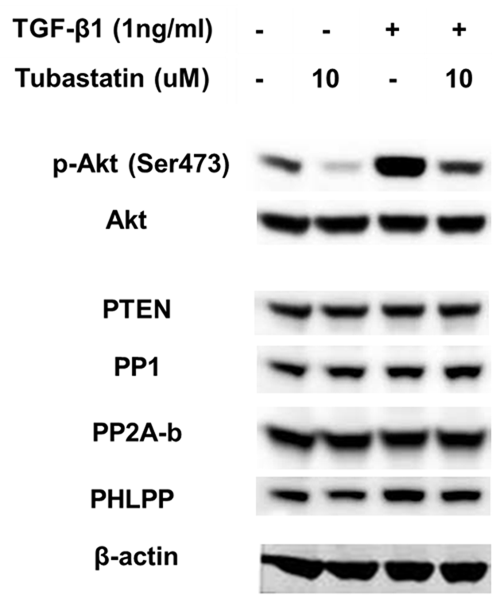

Supplement: S3 Fig — Subconfluent NHLFs were pretreated with Tubastatin for 6 hours and then co-treated with TGF-β1 and Tubastatin for 12 hours. (TIF) [file pone.0186615.s004.tif]
